# Online Group Consultation on Labor Analgesia for Pregnant Women: Is It Feasible?

**DOI:** 10.7759/cureus.51687

**Published:** 2024-01-05

**Authors:** Ana Sofia Tomás, Raquel M Dias, Hermina Cabido, Catarina Nunes, Paulo Lemos

**Affiliations:** 1 Department of Anaesthesiology, Centro Hospitalar Universitário de Santo António, Porto, PRT; 2 Department of Science and Technology, Universidade Aberta, Lisboa, PRT

**Keywords:** analgesia during labor, perioperative care, online consultation, obstetric anaesthesia, e-health

## Abstract

Introduction: Our department of anesthesiology has been conducting weekly, for several years, a group consultation to educate childbearing people about labor analgesia. The emergence of the COVID-19 pandemic forced an adaptation to a virtual consultation format. Since there are no studies about online group consultation on labor analgesia in order to understand its role, an anonymous questionnaire was created and applied. The objective was to evaluate this new consultation format, namely the ease of access, usefulness of the content provided, and its impact on the satisfaction and experience of childbirth.

Materials and methods: An observational prospective study was conducted. A questionnaire was sent by e-mail after childbirth to all childbearing people participating in the online consultation from January 20, 2021, to March 2, 2022. SPSS Statistics version 28.0 (IBM Corp. Released 2021. IBM SPSS Statistics for Windows, Version 28.0. Armonk, NY: IBM Corp) was used for statistical analysis. Internal consistency was analyzed using Cronbach’s alpha.

Results: A total of 563 participants were eligible, and 404 (71.8%) completed questionnaires were analyzed. A few technical problems were reported. The participants considered their privacy respected, and more than 90% were satisfied with the content of the online consultation, the opportunity to pose questions, and the help managing expectations. Considering face-to-face consultation, 89.6% of patients considered the online format an effective alternative, 63.2% believed it could replace the old model, and 96.3% would recommend it.

Conclusions: Our study demonstrates that online consultation on labor analgesia was a good strategy during the COVID-19 pandemic and has the potential to be used in this format in the future.

## Introduction

The use of online consultation has been implemented in several countries. The e-Health concept refers to the integration of medical services and information over the Internet and mobile technologies, such as computers, mobile phones, tablets, and other wireless devices [1]. This growing concept leverages new technologies and has been applied in rural communities, spanning across all specialties [2-4]. Recently, the COVID-19 pandemic has accelerated the adoption of e-Health, prompting anesthesiologists to develop telemedicine programs that enhance care delivery while minimizing exposure risks for patients [5-7].

For several years, our anesthesiology department has conducted a weekly group consultation on labor analgesia for pregnant women during the third trimester. The goal was to explain available labor analgesia techniques, including possible complications and expectation management. Originally conducted in person, the consultation transitioned to a virtual format through a platform for video and audio conferencing due to the COVID-19 pandemic, ensuring clinical safety.

The online labor analgesia consultation, lasting approximately 90 minutes, is led by an anesthesiologist. It includes an eight-minute video presentation depicting different labor stages, followed by a 40-minute slide presentation explaining labor pain, available analgesia techniques, and potential complications. Participants can ask questions during and after the presentations, with the option to email the anesthesiologist for specific concerns answered within 48 hours. Face-to-face consultations are scheduled upon request. An email invitation with a link to a platform is sent the day before the consultation, along with a tutorial to assist participants with platform access.

While studies on online consultation by anesthesiologists in obstetric populations exist, they mainly focus on the perioperative evaluation of high-risk women, lacking specific research on online group consultations for labor analgesia [8-11].

To comprehend the role of online group consultation, specifically its ease of access, the utility of the content provided, and its influence on satisfaction and childbirth experience, we developed an anonymous questionnaire. Our primary objective, through the questionnaire, was to initially assess the feasibility of the consultation via the conferencing platform and subsequently evaluate satisfaction related to the childbirth experience.

## Materials and methods

We conducted an observational prospective study, approved by the hospital's research department, the ethics committee of Centro Hospitalar Universitário do Porto (research project number: 2021.131 (107-DEFI/110-CE)), and the board of directors.

A questionnaire created by the authors assessed the adequacy, realism, and utility of the online consultation format post-COVID-19 (Appendices section). The eligible population comprised all pregnant women participating in the online consultation from January 20, 2021, to March 2, 2022. Referrals for labor analgesia consultation were primarily made by nurses, with some referrals also coming from obstetricians. Individuals could also voluntarily propose themselves for the consultation, as it was advertised in informational materials at the hospital.

The questionnaire was distributed via email to the eligible population between one week and three months after childbirth. It was voluntary and anonymous, crafted and administered in Portuguese, a language understood by all participants. The questionnaire included a brief explanation of the study's purpose and sought informed consent. Comprising 21 questions, the questionnaire was divided into four subgroups: demographics, obstetric history, last birth history, and labor analgesia consultation. Two questions were presented in a 5-point Likert scale format, totaling 15 items. Additionally, three questions allowed for free-text comments on labor experience, newborn issues, and suggestions. The questionnaire concluded by assessing overall satisfaction with childbirth on a scale of 0 to 10. Only completed questionnaires were analyzed. The results presented focus on the labor analgesia section and demographic data.

Statistical analysis utilized SPSS Statistics version 28.0 (IBM Corp. Released 2021. IBM SPSS Statistics for Windows, Version 28.0. Armonk, NY: IBM Corp), summarizing all variables through descriptive statistics. Internal consistency was assessed using Cronbach’s alpha, considering the 15 items forming the two Likert scale questions.

## Results

A total of 563 women were eligible for the study, representing 20% of annual births at our institution. Of these, 404 (71.8%) completed the questionnaire, 81 (14.4%) started but did not finish the questionnaire, and in four (0.7%) cases, the email was returned. Only the 404 complete questionnaires were considered for analysis. The internal consistency analysis for items on the Likert scale yielded a Cronbach’s alpha of 0.738.

**Figure 1:**
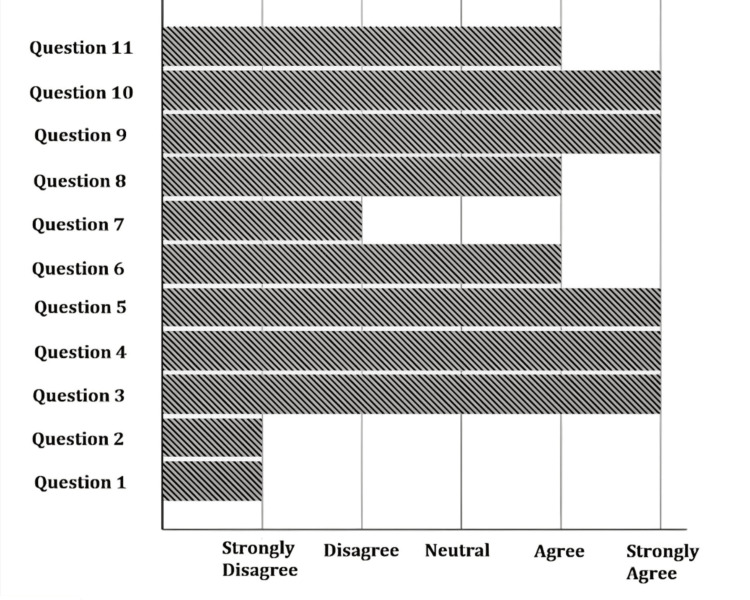
Median values of the questions in Table 2

The age of the women ranged between 20 and 46 years old, with a median of 33 years. Table 1 summarizes demographic data. Women participated in the consultation using a computer (60.4%), mobile phone (31.4%), or tablet (7.4%), and 0.7% of them did not remember the device used. Table 2 summarizes the answers (according to the Likert scale) to the questions about access, content, and privacy of the online consultation. When asked about the appropriateness of the consultation duration (90 minutes), three (0.7%) considered it too short, 19 (4.7%) short, 364 (90.1%) adequate, and 18 (4.5%) lengthy. The answers to questions about the consultation are summarized in Figure 1.

**Table 1:** Descriptive statistics of body mass index and educational level

Body mass index	n (%)
*≤*18.5	9 (2.2%)
18.6-24.9	189 (46.8%)
25-29.9	141 (34.9%)
30-34.9	45 (11.1%)
35-39.9	13 (3.3%)
*≥*40	7 (1.7%)
Median	25.1
Education level	n (%)
Less than middle school	19 (4.7%)
Less than high school	121 (30%)
College graduate	167 (41.3%)
Master’s degree	91 (22.5%)
Doctorate	6 (1.5%)

**Table 2:** Answers to the questions regarding the online consultation (Likert scale)

Questions	Strongly disagree n (%)	Disagree n (%)	Neutral n (%)	Agree n (%)	Strongly agree n (%)
1 - I had difficulty in accessing the online consultation	333 (82.4%)	56 (13.9%)	3 (0.8%)	5 (1.2%)	7 (1.7%)
2 - I had difficulty in watching and hearing the shared content	323 (79.9%)	65 (16.1%)	3 (0.8%)	8 (2%)	5 (1.2%)
3 - The audiovisual content (video and PowerPoint presentation) exposed was enlightening	9 (2.2%)	2 (0.5%)	6 (1.5%)	132 (32.7%)	255 (63.1%)
4 - I had the opportunity to expose and clarify my doubts	1 (0.2%)	3 (0.8%)	8 (2%)	106 (26.2%)	286 (70.8%)
5 - The labor analgesia consultation helps in managing expectations and adapts to reality	6 (1.5%)	16 (4%)	18 (4.5%)	152 (37.6%)	212 (52.4%)
6- The labor analgesia consultation allowed the sharing of experiences with other pregnant women	13 (3.2%)	15 (3.7%)	115 (28.5%)	133 (32.9%)	128 (31.7%)
7 - The labor analgesia consultation as presented does not allow for maintaining the required and desired privacy in the transmission of clinical information	122 (30.2%)	104 (25.7%)	68 (16.8%)	73 (18.1%)	37 (9.2%)
8 - If needed, I felt that an alternative communication would be provided to guarantee the privacy of the doctor-patient relationship	12 (3%)	22 (5.4%)	58 (14.4%)	125 (30.9%)	187 (46.3%)
9 - I would recommend the labor analgesia consultation to pregnant family members and/or friends	2 (0.5%)	3 (0.8%)	10 (2.5%)	116 (28.7%)	273 (67.6%)
10 - The online consultation model, imposed by the COVID-19 pandemic, is a practical and effective alternative to face-to-face consultation	8 (2%)	16 (4%)	18 (4.5%)	143 (35.3%)	219 (54.2%)
11 - After the resolution of the pandemic situation, the consultation via the conferencing platform can replace the face-to-face format	39 (9.7%)	53 (13.1%)	57 (14.1%)	103 (25.5%)	152 (37.6%)

## Discussion

Over the past decade, the use of telemedicine for patient evaluation and treatment has grown, with healthcare systems increasingly incorporating telemedicine visits across specialties [12]. Anesthesiologists, traditionally with limited experience in telemedicine for perioperative care, have faced unprecedented challenges due to COVID-19, pushing the boundaries of medicine and necessitating solutions to problems that previously impeded rapid progress [13]. The curtailment of the traditional face-to-face model of preoperative assessment led to the exploration of telemedicine.

Telemedicine has the potential to reduce travel time, financial burden, and absence from work, improve accessibility in a secure environment with a more pleasant waiting time, and increase patient satisfaction by enabling anesthesia specialists to remotely consult on patients using technologies such as video communication.

While anesthesiologists play a vital role in perinatal evaluation, coordination, and planning for optimal peripartum birth care, the field of obstetric anesthesia often involves parturients being evaluated by their anesthesiologist only on the day of labor admission or just before epidural placement. Prenatal anesthetic evaluation consultations are typically reserved for high-risk pregnancies or cases with previous anesthetic complications [10,14].

The labor analgesia consultation, performed as a group consultation at our institution for several years, aims to provide information to all pregnant women during the third trimester. Early consultation enables the provision of information about available analgesia techniques, addressing questions, and managing expectations. The rationale for this project was to reduce in-person attendance during COVID-19 restrictions while maintaining effective and safe group online consultations. Telemedicine shows great potential in anesthesiology, given the rapid growth of technology and equipment. However, like any new technology in the practice of medicine, it must first be tested and validated.

To our knowledge, guidelines for the use of telemedicine in the preanesthetic evaluation setting have not been developed by the European Society of Anaesthesiology and Intensive Care or other European governance bodies. The U.S. Department of Health and Human Services called upon the National Quality Forum (NQF) to convene a multistakeholder committee to recommend various methods to measure the use of telehealth as a means of providing care. The NQF defined essential categories for measuring telehealth in birth care, including access to care, the financial impact on women and their care providers, women and clinician experience, and the effectiveness of clinical and operational systems. Among these, the NQF suggested six priority areas: travel, timeliness of care, actionable information, the impact of telehealth in providing evidence-based practices, patient empowerment, and care coordination. These NQF recommendations can serve as a guide in creating metrics for the impact of technology in anesthesiology and perioperative medicine [15].

In order to implement this new consultation format, we conducted a literature review on online anesthesia consultation in the obstetric population and found no reports in this format. The first reported use of telemedicine in anesthesiology was in 2004 when Wong et al. reported a case series where 10 patients underwent preoperative evaluation using a viewing screen equipped with a mounted camera operated by a nurse at a remote facility [4]. A limited number of studies have consistently compared the accuracy of online and in-person assessments and exam consultations. In a prospective study conducted by Applegate et al., 155 patients were randomized to an in-person or telemedicine preoperative anesthesia evaluation. The authors concluded that, compared with in-person consultations, telemedicine physical examinations were as accurate, and telemedicine documentation was considered superior. There was a high rating of satisfaction among both patients and providers for both telemedicine and in-person arms [16]. In 2013, Roberts et al., in the remote areas of the Northern Territory of Australia, investigated the patient's perception of virtual preoperative anesthesia assessment using a 10-item, 5-point scale questionnaire, evaluating perception in four domains: technical quality, perceived efficacy, affective experience, and patient preference. They concluded that the study confirmed the acceptability of telemedicine in the remote assessment of preoperative patients, with positive perceptions in all four domains [17].

In a more recent study with 2805 women conducted by Popivanov et al. during the COVID-19 pandemic, the aim was to develop, implement, and evaluate a high-quality preoperative anesthetic evaluation process in obstetrics and gynecology, comparing face-to-face consultation with online consultation, with positive outcomes for the latter [18]. Morau et al. conducted a study, also during the COVID-19 pandemic, with a small sample size, aiming to study the technical feasibility and patient experience of teleconsultation for preoperative assessment using a 4-point Likert scale questionnaire. They concluded that despite technical problems reported by 24% of the participants, the vast majority gave a high score for information about anesthesia, comfort of teleconsultation, and confidence in the transmitted information [6].

In our study, 583 pregnant women participated in the group's online consultation, and out of these, 404 completed the questionnaire, resulting in a complete response rate of more than 70%, consistent with the response rates in other published studies on preoperative anesthesia online consultation [6,17,19]. Regarding responses about access to the consultation, 2.9% (n=12) reported having had problems accessing the consultation via the conferencing platform, despite the explanatory tutorial provided on how to access it, and 3.2% (n=13) experienced difficulty in seeing/listening to the contents. Morau et al. also reported technical problems (no connection, no picture, and no sound), but in 24% of cases [6]. The written tutorial sent along with the access link can help reduce technical issues related to platform connections, which may partly justify the lower percentage of technical problems reported in our study.

Regarding the content of the online consultation, more than 90% of women considered the content to be enlightening, appropriate for managing labor expectations, had the opportunity to express their doubts, and would recommend consultation on labor analgesia to pregnant family members or friends.

The implementation of the online group consultation on labor analgesia seemed to have a positive effect, as 89.6% of women considered online consultation an effective alternative to face-to-face consultation, and 63.2% believed that online consultation could replace face-to-face consultation.

Figure 1 summarizes the answers to Likert scale questions about the consultation. It is clear that most participants did not have technical problems (more than 80%, questions 1 and 2, Table 2) and considered their privacy respected (question 7). For the remaining questions, the majority agreed or strongly agreed, demonstrating the participants' consensus on the adequacy of the content, the opportunity to pose questions, assistance in managing expectations, experience sharing, and an alternative for exposure to personal issues in case of need. Consultation was considered a good alternative to face-to-face consultation during the COVID-19 pandemic and a good format for future consultations.

Popivanov et al. also reported that more than 90% of patients agreed that consultation time was sufficient, as in our study, and that they felt listened to; online consultation saved them time and money, and they would be happy to use online consultation in the future [18]. Previous studies had reported dissatisfaction related to limited internet access, poor email usage, and concerns with privacy and data security [20-22]. In our study, the number of women who want to participate in online group consultations is high. This can be partially explained by the higher level of education of our population compared to the Portuguese population, where only 19.8% have higher education versus 65.3% in our study [23]. Additionally, other contributing factors may include being young women, having concerns about labor, and residing in a metropolitan area with easy access to electronic devices and an internet network that allows convenient access to online consultation.

Our study has some limitations and biases. The study contains some design imperfections that may contribute to its limited validity. Considering certain constraints, there was considerable variability in the time gap between childbirth and questionnaire delivery, posing a potential risk of retention or memory bias. The generalizability of this study’s findings is limited by its specific structure. The contribution of our study to evidence-based medicine would be greater if we had compared the experience of online consultation versus face-to-face consultation; however, this was not possible given the pandemic context.

## Conclusions

We believe that the group online consultation on labor analgesia was a beneficial initiative during the COVID-19 pandemic and has the potential to continue in this format in the post-pandemic setting. The use of telemedicine within anesthesiology is a relatively new concept compared to other specialties, but it is gaining prominence in practice, and its utilization in the preoperative and postoperative periods is expected to grow in the future. Guidelines for the use of telemedicine must be developed by European governance bodies, and robust outcomes research is necessary to assess the clinical effectiveness of these new technologies for patient care.

## Figures and Tables

**Figure 2:**
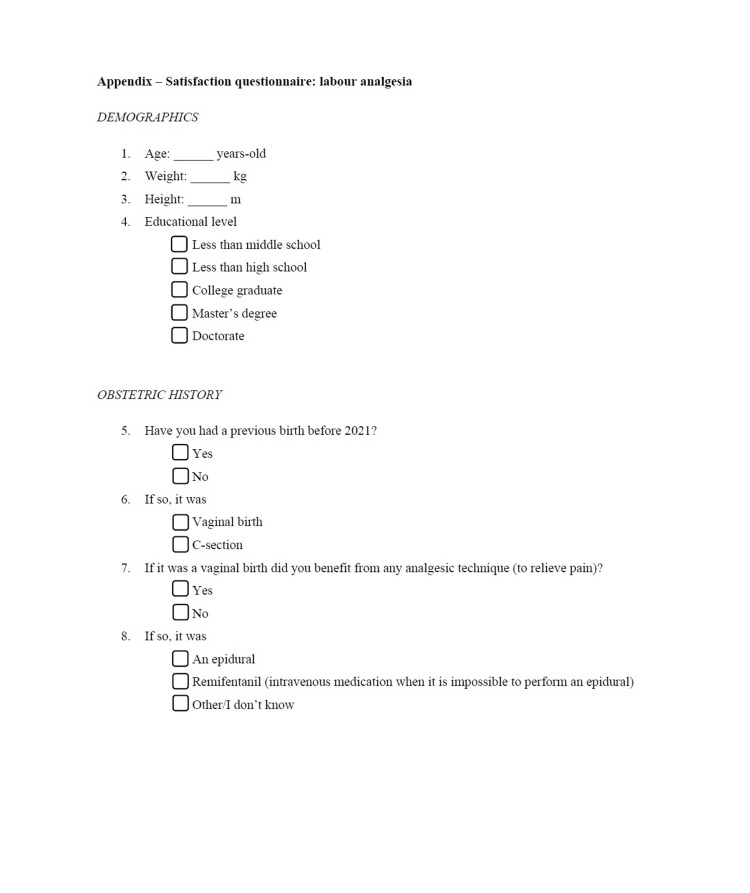
Satisfaction questionnaire: labor analgesia (part 1)

**Figure 3:**
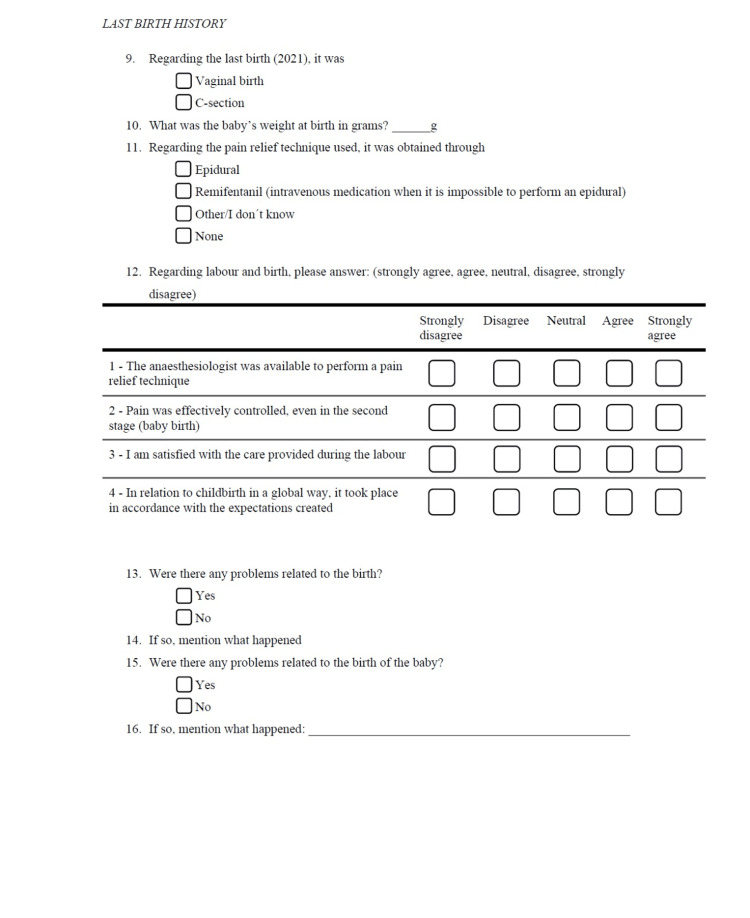
Satisfaction questionnaire: labor analgesia (part 2)

**Figure 4:**
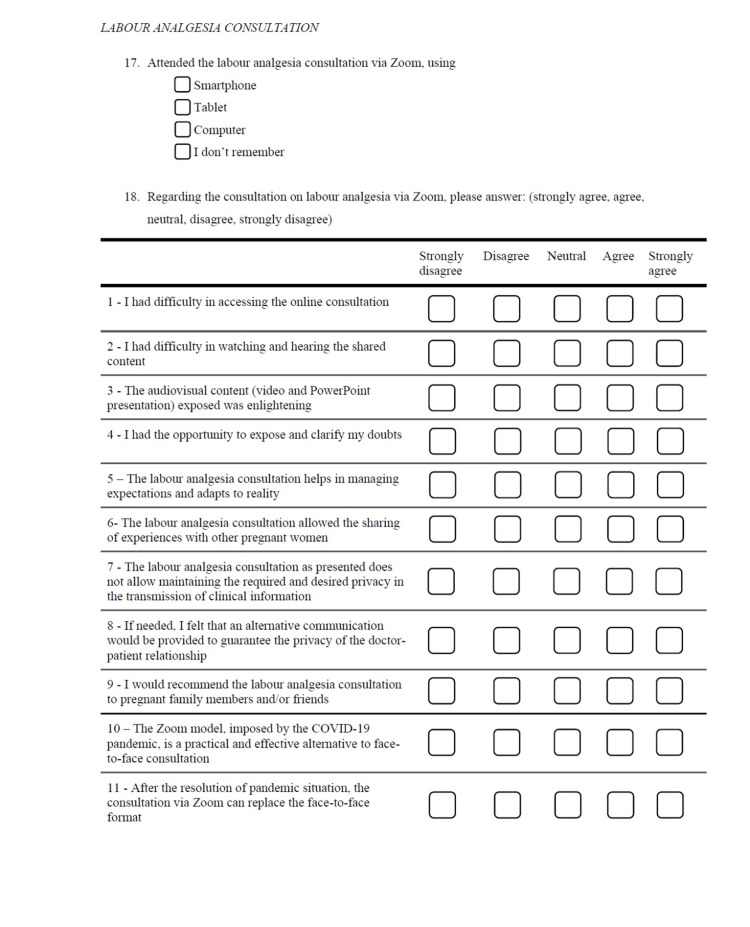
Satisfaction questionnaire: labor analgesia (part 3)

**Figure 5:**
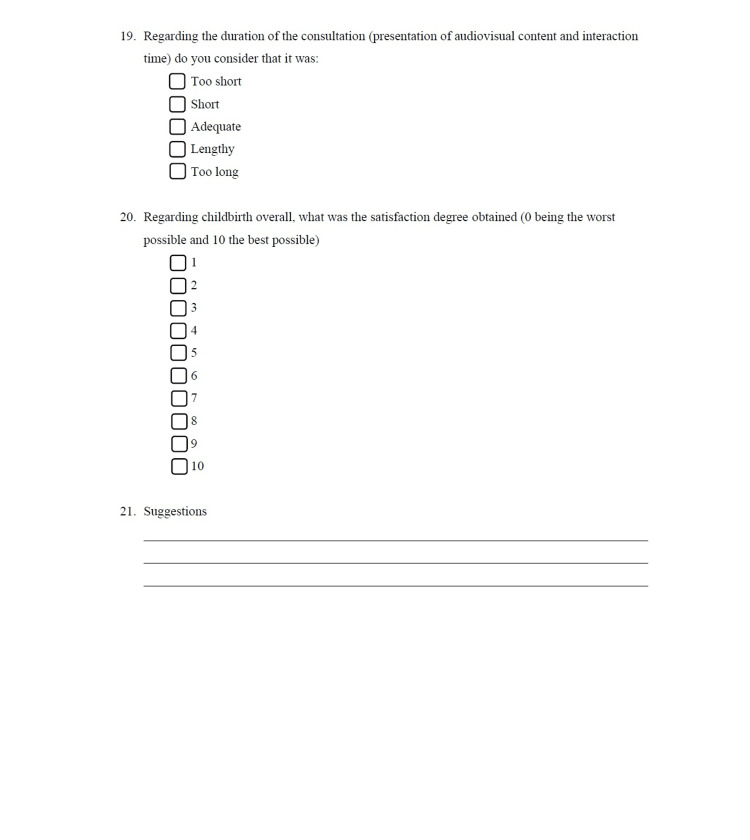
Satisfaction questionnaire: labor analgesia (part 4)
